# Cerebellar Prediction and Feeding Behaviour

**DOI:** 10.1007/s12311-022-01476-3

**Published:** 2022-09-19

**Authors:** Cristiana I. Iosif, Zafar I. Bashir, Richard Apps, Jasmine Pickford

**Affiliations:** School of Physiology, Pharmacology and Neuroscience, University of Bristol, Biomedical Sciences Building, University Walk, Bristol, BS8 1TD UK

**Keywords:** Cerebellum, Feeding behaviour, Hunger, Satiation, Reward

## Abstract

Given the importance of the cerebellum in controlling movements, it might be expected that its main role in eating would be the control of motor elements such as chewing and swallowing. Whilst such functions are clearly important, there is more to eating than these actions, and more to the cerebellum than motor control. This review will present evidence that the cerebellum contributes to homeostatic, motor, rewarding and affective aspects of food consumption.

Prediction and feedback underlie many elements of eating, as food consumption is influenced by expectation. For example, circadian clocks cause hunger in anticipation of a meal, and food consumption causes feedback signals which induce satiety. Similarly, the sight and smell of food generate an expectation of what that food will taste like, and its actual taste will generate an internal reward value which will be compared to that expectation. Cerebellar learning is widely thought to involve feed-forward predictions to compare expected outcomes to sensory feedback. We therefore propose that the overarching role of the cerebellum in eating is to respond to prediction errors arising across the homeostatic, motor, cognitive, and affective domains.

## Introduction

The cerebellum is the largest sensorimotor structure in the brain and has traditionally been associated with motor control and the coordination of voluntary movements, balance, and posture [1, 2, 3]. The cerebellum is now known to contribute to a wide range of behaviours extending beyond motor control including higher-order functions such as cognitive processing [4, 5, 6], reward signalling [4, 7, 8], and affective processing [9, 10], as well as fundamental functions including visceral control [4, 7, 8] and survival behaviours [11, 12, 13, 14]. Contributions to such an array of functions mean the cerebellum is well placed to act as a hub for processing multi-modal information involved in mediating complex behaviours.

One such complex behaviour is eating—an essential function to provide the energy and nutrition required to live [15, 16, 17]. But we do not just eat to survive, we also eat for pleasure [18, 19, 20]. Hunger and hedonic desire provide motivation to seek food, movement is required to locate and consume food, and then digestive processes break down food in the gut and provide feedback to the brain to regulate the amount eaten [21, 22]. Eating can also be influenced by emotional and pathological states, such as overeating in obesity and undereating in anorexia nervosa [23, 24], emphasising that food consumption has both homeostatic and higher-order elements.

This review will provide a brief introduction to the cerebellar organisation to inform the following discussion of its role in (1) motor aspects of eating, (2) homeostatic and hedonic elements of appetite, (3) reward processing in relation to healthy and disordered eating, and (4) affective processing related to appetite and reward. The cerebellum is implicated in many of the processes involved in food consumption, and its activity and network connectivity are altered in both overeating (obesity) and undereating (anorexia nervosa) disorders which also span the above domains. We therefore propose that the cerebellum serves as a central regulator of information processing across the homeostatic, motor, cognitive and affective domains. More specifically, given that the cerebellum is known to create predictive representations of the environment and that eating behaviours are underpinned by expectation, we will present evidence that the universal role of the cerebellum in eating is to generate behaviourally relevant responses to prediction errors.

## Overview of Cerebellar Structure

**Fig. 1:**
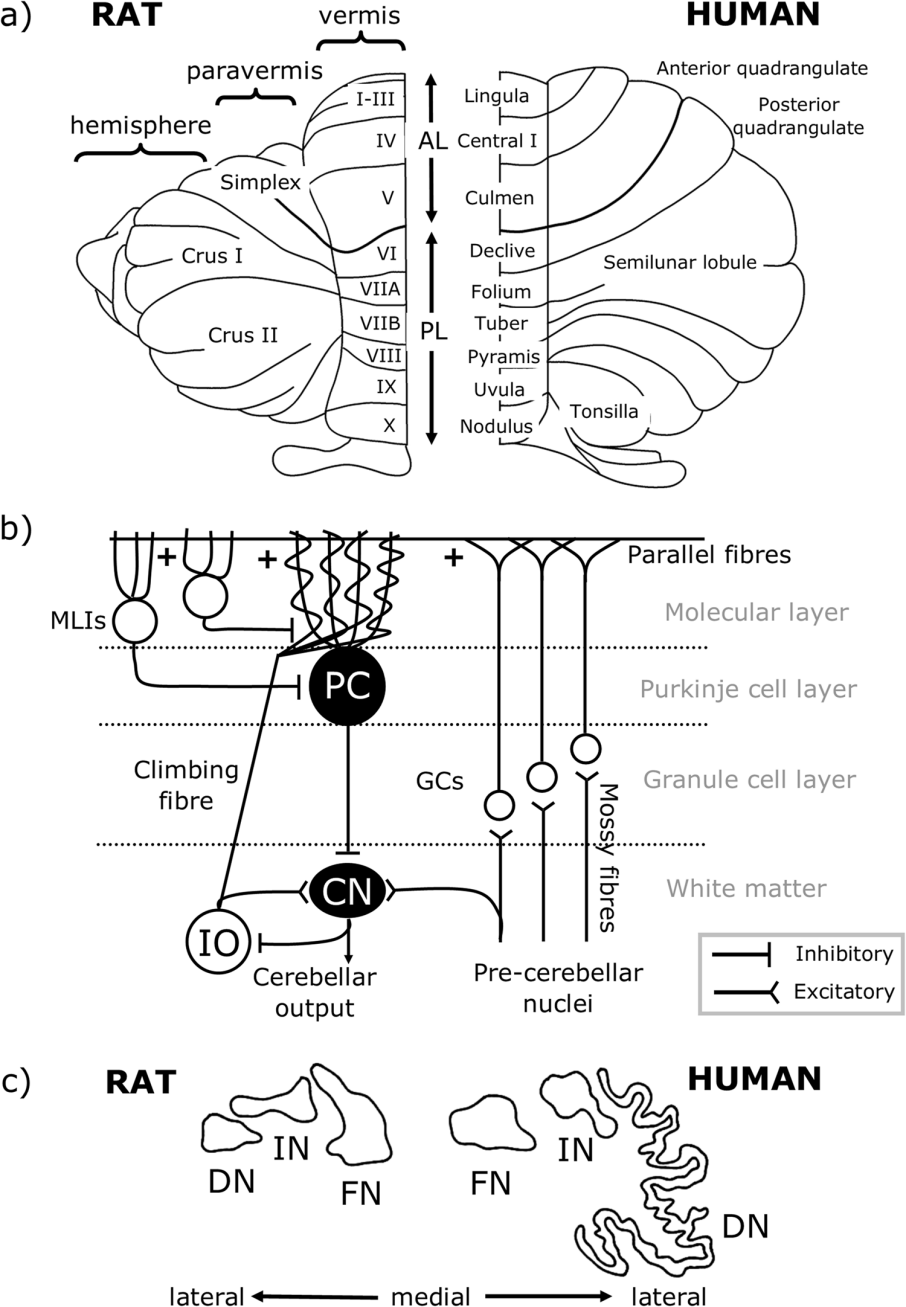
Cerebellar anatomical organisation. **a** Dorsal view of the rat (left) and human (right) cerebellum. There are three main longitudinal compartments of the cerebellar cortex, from medial to lateral the vermis, paravermis and hemisphere. AL, anterior lobe; PL, posterior lobe. **b** Simplified cerebellar circuitry. Inputs to the cerebellum are from mossy fibres of various pre-cerebellar nuclei and climbing fibres of the inferior olive (IO), both of which are glutamatergic. Mossy fibres synapse onto granule cells (GCs) which form bifurcating axons, known as parallel fibres, targeting Purkinje cell (PC) dendrites, and climbing fibres synapse onto PC dendrites directly. Both mossy fibres and climbing fibres also form collaterals targeting neurons of the cerebellar nuclei (CN). PCs are the sole output neuron of the cerebellar cortex, and these GABAergic neurons target neurons of the CN which form cerebellar output. Several types of interneurons also act within the cerebellar cortex, including molecular layer interneurons (MLIs), not all of which are shown. **c** Outlines of the rat (left) and human (right) cerebellar nuclei. The vermis, paravermis and hemispheres of the cerebellar cortex project to the fastigial nuclei (FN, also known as medial nuclei), interpositus nuclei (IN) and dentate nuclei (DN, also known as lateral nuclei), respectively. Scaled so that FN is a similar size in both species. Adapted from Altman and Bayer [26]

To understand cerebellar function, it is necessary to appreciate its basic anatomical organisation. At a macroscale the cerebellar cortex has three rostro-caudally oriented longitudinal divisions: from medial to lateral on each side of the cerebellar midline these are the vermis, paravermis and hemisphere (Fig. 1a). The cerebellar cortex is intricately folded and has a tri-laminar structure comprised of the granule cell layer, Purkinje cell layer, and molecular layer, the circuitry of which is summarised in Fig. 1b. The cells within these cortical layers process cerebellar inputs and Purkinje cells form the sole output of the cerebellar cortex, projecting to neurons of the cerebellar nuclei (Fig. 1b), which, in turn, provide the final cerebellar output signal. Purkinje cells located within the vermis, paravermis, and hemispheres project mainly to the fastigial (medial), interpositus and dentate (lateral) nuclei respectively (Fig. 1c) [25].

The medial parts of the cerebellum are the oldest in evolutionary terms, with roles in motor control, proprioception and autonomic functions [27]. In contrast, the hemispheres are more highly developed in higher-order species in line with the expansion of the cerebral cortex [28]. These lateral cerebellar regions are related to goal-directed behaviour, including cognition, due to extensive cerebro-cerebellar connections [29, 30]. The cerebellar nuclei have also expanded disproportionately with evolutionary development; by comparison to fastigial and interpositus, the dentate nucleus is larger and more convoluted in higher species in accordance with an increase in the size of the cerebellar hemispheres from which it receives input (Fig. 1c) [26].

At a finer level of anatomical organisation, the cerebellum contains a series of “modules” consisting of rostrocaudally oriented “zones” of Purkinje cells in the cortex together with the cerebellar nuclear territory that they target, and the inferior olive neurons from which they receive climbing fibre input (for further detail on modules and zones see [31, 32, 33, 34, 35]). These olivo-cerebellar loops are thought to be the basic functional units of the cerebellum. The function of each is thought to be dictated by its input and output connectivity with other regions of the brain [32, 33, 36, 37, 38, 39]. The modular organisation of the cerebellum should therefore be taken into account when considering the contributions of individual cerebellar regions to behaviour, including eating.

An additional important consideration is that the cerebellum has been proposed to act as a ‘prediction machine’ [40, 41], participating in the formation and updating of internal models which allow ongoing behaviours to be modified based on prior experience (Wolpert et al. [42]). As such, the cerebellum is likely to control behaviour by generating predictions about future behavioural outcomes which are updated based on the comparison of actual and expected outcomes [40]. Similar predictive mechanisms may apply across multiple domains via cerebellar connections with a multitude of brain regions, allowing the cerebellum to optimise many types of behaviour, including those involved in eating.

## Cerebellar Contributions to Motor Control of Eating

Perhaps the most obvious role of the cerebellum in eating is its contribution to motor behaviour. Aside from its involvement in the movement required to locate food and bring it to the mouth, the cerebellum contributes to the two main motor components of consuming food: mastication (chewing) and swallowing [43]. Cerebellar injuries, stroke, and ataxia are associated with difficulties swallowing (dysphagia) and chewing [44, 45, 46, 47, 48], demonstrating the importance of an intact cerebellum in the physical ability to eat. This section will discuss the role of the cerebellum in both the voluntary (mastication and initiation of swallowing) and involuntary (passive swallowing) motor aspects of food ingestion.

### Cerebellum in Mastication

Mastication is voluntary and recruits different groups of facial and neck muscles depending on the difficulty encountered at food breakdown [22]. The cerebellum indirectly innervates facial muscles via the red nucleus [49], a key component of the lateral descending motor system (for review see [50]).

Functional magnetic resonance imaging (fMRI) is commonly used to non-invasively assess brain activity, particularly in humans. Increased regional blood flow is linked to neuronal activation, and the changes in levels of oxygenated and deoxygenated blood can be detected as a blood oxygen level-dependent (BOLD) contrast signal which indicates whether a brain region is showing relatively increased or decreased activity [51]. Such imaging studies in humans have shown that most regions of the posterior cerebellum change their activity in relation to chewing, with activation of the anterior cerebellum and of lobules V, VI, VIII, and IX of the posterior cerebellum during chewing and associated facial movements [52, 53, 54, 55, 56, 57]. Co-activation of the cerebellum (lobule VI), thalamus and supplementary motor areas in the cerebral cortex during chewing and clenching of the jaw indicate that the cerebellum may modulate masticatory activity via cerebello-thalamo-cerebral connections [54, 56, 58, 59].

Internal and external cerebellar functional connectivity is also increased during chewing; fMRI has shown enhancements in both inter-hemispheric connectivity within the cerebellum and with other brain regions including sensorimotor cortices, left temporal gyrus and left cingulate cortex [60]. The pattern of cerebral connections suggests the cerebellum is involved in the motor planning element of mastication, in line with theories of cerebellar prediction and anticipatory activity [60, 61]. Furthermore, patients who adjust their chewing movements after structural changes in the dental arch show in fMRI studies an increased involvement of the cerebellum [57], supporting the potential involvement of the cerebellum in chewing pattern generation [52]. This is consistent with the cerebellum containing an internal model related to chewing, which is updated when oral modifications change the most efficient form of chewing. Taken together, imaging studies in humans therefore suggest a role for the cerebellum in the motor activity, planning and updating of internal models related to mastication.

### Cerebellum in Swallowing

Swallowing can be divided into three phases: (i) the oral preparatory phase, comprising of the formation of the bolus and voluntary guidance towards the larynx; (ii) the pharyngeal transfer phase, where a series of reflexes induce involuntary closure of the epiglottis and guidance of the bolus towards the oesophagus; and (iii) the oesophageal transport phase, when the bolus is transported towards the stomach by synchronised contraction and relaxation of the circular muscles [21]. Based on anatomical, physiological, imaging, and clinical evidence, the cerebellum has an established role in all phases of swallowing (for a more extensive review, see [48]). For example, fMRI studies in humans have shown cerebellar activation during swallowing, particularly within the left-hand side of the cerebellum (around lobule VI, vermal culmen and pyramis) [62, 63, 64].

The cerebellum has reciprocal connections with cranial nerves involved in mediating pharyngeal and oesophageal phases of swallowing; typically, these nerves have a sensory component with projections to the cerebellum, and a motor component which controls and coordinates pharyngeal and oesophageal muscle activity according to feedback [65]. Somatotopic representations of the facial area are found across the cerebellar cortex [66]. In particular, the tongue is a critical component of the voluntary phase of swallowing [67], and there is a somatotopic representation of the tongue across lobules VII and VIII of the cerebellar cortex in humans [62]. A perioral representation with fractured somatotopic organisation has also been demonstrated in the cerebellar hemisphere granule cell layer in other species, including rodents [68, 69], suggesting conservation of function across species.

**Fig. 2:**
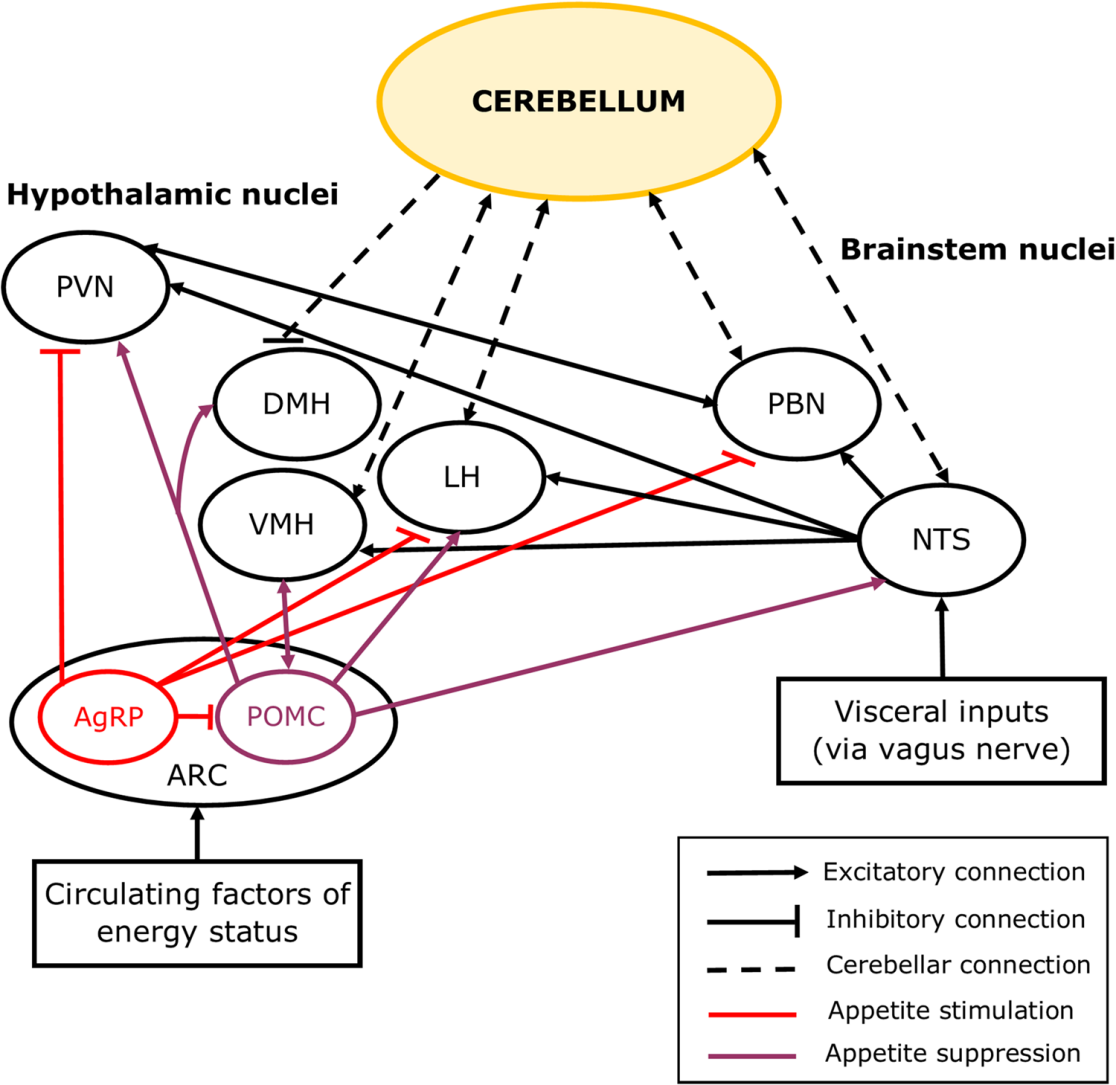
Simplified diagram showing cerebellar connections with feeding circuits in the brain. The hypothalamic nuclei are central to a network of brain regions which regulate appetite. Distinct subtypes of neurons in the arcuate (ARC) nucleus of the hypothalamus are involved in the initiation (AgRP neurons) or cessation of food consumption (POMC neurons) via their inputs to the other hypothalamic nuclei including the paraventricular hypothalamic nucleus (PVN), ventromedial hypothalamic nucleus (VMH), lateral hypothalamic nucleus (LH), and dorsomedial hypothalamic nucleus (DMH). Short-term appetite regulation involves the parabrachial nucleus (PBN) and the solitary tract nucleus (NTS) of the brainstem, which respond to feedback from the gut and form connections with the hypothalamus to initiate satiation. The cerebellum has reciprocal connections with the VMH, LH, PBN and NTS, and sends inhibitory projections to the DMH

The cerebellum also has reciprocal connections with the nucleus of the solitary tract (NTS, Fig. 2), which integrates visceral information from cranial nerves, and amongst other functions, initiates the voluntary phase of swallowing [70]. The NTS has direct projections to the cerebellar cortex, particularly the vermis, and receives inputs from the medial nucleus of the cerebellum [70, 71], which relays output from the vermis, thereby forming a reciprocal NTS-cerebellar circuit. Transcranial magnetic stimulation of the cerebellum in humans interferes with swallowing, indicating that the cerebellum, like the NTS, plays a pivotal role in coordinating voluntary swallowing [72].

Physiological studies have also provided direct evidence for a role of the cerebellum in swallowing. In the awake cat and rat, electrical stimulation of the cerebellar vermis and medial nucleus elicits swallowing, gnawing and grooming behaviours [73, 74]. In addition, cerebellectomy in anaesthetised cats reduces motor recruitment in the pharyngeal-oesophageal area [75].

Together, the available evidence from human and animal studies demonstrates that the cerebellum is involved in all of the key stages of food ingestion, from voluntary preparatory and transfer phases of swallowing [63, 64], to the involuntary transport phase [73, 76]. Chewing and swallowing are stereotypical, repetitive behaviours and therefore predictable, making them well suited to being represented by cerebellar internal models. In the future, the advance of cell-specific genetic methods to manipulate cerebellar circuitry in animal models will help reveal the mechanisms and precise networks by which the cerebellum mediates these motor behaviours.

## Cerebellar Contributions to Appetite Control

Appetite control is a complex process balancing feedback information from energy stores and reward centres relating to hunger and desire to eat, and feed-forward information from the body’s internal clock in anticipation of mealtimes. Although not widely recognised, the cerebellum contributes to these processes through its connections with well-established brain circuits involved in feeding behaviour.

### Cerebellum in Hunger and Satiation

Homeostatic mechanisms associated with eating maintain energy stores by either increasing or decreasing food intake to match energy requirements [15]. Food can also be rewarding, so hedonic mechanisms can initiate or limit food intake and can sometimes overrule homeostatic mechanisms [19]. For example, hunger produces an unpleasant sensation in response to low energy stores that drives appetitive behaviours to replenish the energy stores [15, 17, 77], but the sensation of hunger can also be induced by exposure to food cues and cravings [19]. The hypothalamus is the central regulator of appetite and has reciprocal connections with the cerebellum [15, 16, 19, 78, 79, 80]. Both the cerebellum and hypothalamus interact with other brain structures regulating homeostatic and hedonic appetite (Fig. 2, section The Cerebellum in the Homeostatic and Hedonic Appetite Regulation Circuitry).

#### Cerebellar Activation in Hunger and Satiation

Cerebellar activity changes with hunger state, reflecting its contributions to the feeding circuitry described above. For example, imaging studies in humans using positron emission tomography (PET) in combination with injection of radioactive water found increased regional blood flow in the cerebellum following a 36-h fast in healthy-weight participants, particularly within the anterior and midline vermis [81]. Cerebellar activation during hunger was significantly decreased when satiation was induced via liquid meal ingestion [81, 82] suggesting that the cerebellum may be responsive to nutrient intake, perhaps by responding to one of the circulating factors released during food consumption (e.g. glucose or cholecystokinin) which provide fast-acting signals regulating appetite [83, 84, 85, 86].

A comparison between healthy-weight and overweight participants, using the same PET technique as above, found that a larger area of the anterior-midline cerebellar vermis had decreased regional blood flow when satiation was induced following a 36-h fast in overweight participants [87, 88]. This suggests that the cerebellum functions differently in healthy-weight and overweight participants and raises the possibility that modified cerebellar processing plays a role in the pathophysiology of overeating disorders.

During food consumption, stomach stretch is communicated to the brain via the vagus nerve, providing feedback from the gut that food ingestion has begun and serving as a satiation signal [89]. Imaging studies in humans have reported increased activation in the cerebellar uvula (vermal lobule IX) in response to stomach stretch [90]. The strength of the BOLD signal in response to stomach stretch increased linearly with body mass index [90] indicating that the responsiveness of the cerebellum to mechanical, and possibly chemical, feedback from the gut increases with body weight.

Whilst the anterior midline cerebellar vermis is activated during hunger, posterior areas of the vermis are responsive to feedback generated by food ingestion [81, 82, 87, 88, 90]. As outlined in the introduction, this may be related to differential connectivity of cerebellar regions, and food anticipatory processes may vary depending on the size of the metabolic store.

#### The Cerebellum in the Homeostatic and Hedonic Appetite Regulation Circuitry

The hypothalamic nuclei involved in appetite regulation form an intricate network [15, 19, 91]. In brief, the lateral hypothalamic nucleus (LH) is widely considered to be a hub for regulating homeostatic and hedonic feeding [19]. The LH integrates information from the arcuate nucleus of the hypothalamus (ARC, Fig. 2), which contains both hunger-related neurons (expressing Agouti-related protein, AgRP) and satiation-related neurons (expressing pro-opiomelanocortin, POMC). AgRP neurons project to the paraventricular nucleus of the hypothalamus (PVN), LH and brainstem parabrachial nucleus (PBN) in response to lowering levels of the carbohydrate energy store, and POMC neurons mainly project to the PVN, but also to the dorsal medial hypothalamic nucleus (DMH), LH, ventromedial hypothalamic nucleus (VMH), and NTS, in response to nutrient ingestion (Fig. 2) [15, 17, 92, 93, 94]. 

Whilst the cerebellum has generally been overlooked as a component of this homeostatic feeding circuit, anatomical evidence from a range of animal species (including rat, cat, and monkey) has demonstrated a direct reciprocal connection between the cerebellum and hypothalamic nuclei including the DMH, VMH and LH (Fig. 2) [83, 95, 96]. Activation of lateral cerebellar nuclei neurons in mice reduced AgRP neuron-mediated food consumption, indicating that the cerebellum can influence homeostatic control of hunger [97]. The cerebellum also forms reciprocal connections with satiety centres in the brainstem; the NTS (see section Cerebellum in Swallowing for roles of this connection in swallowing) [71, 98] and the PBN [99, 100].

Upon food ingestion, feedback from the gut is communicated to the NTS and the brainstem satiation centre in the PBN, which is passed on to hypothalamic nuclei responsible for inducing satiation and meal termination (Fig. 2) [17, 70, 86, 101]. Circulating factors are also released from the gut and accessory organs and are transported across the blood–brain barrier to reach receptors in a number of areas including the brainstem, hypothalamus, amygdala and cerebellum [15, 101, 102, 103, 104].

LH neurons also integrate information from the ventral tegmental area (VTA) and nucleus accumbens (NAc), which are involved in processing food and reward-related cues [105, 106, 107, 108]. More specifically, the dopaminergic systems in the VTA, NAc and the striatum are involved in driving rewarding behaviours and attributing a reward-value to food to prolong feeding [15, 19, 109]. Recently the cerebellum has also been found to signal various elements of reward as further described in the section Cerebellar Reward Processing and Contributions to Over-Eating. In addition, a subclass of glutamatergic neurons in the mouse lateral cerebellar nuclei have been shown to reduce food intake by increasing the release of dopamine from the VTA [97]. When dopamine is released during consumption, the ingested food is given a reward value indicating that the hedonic need for consumption has been met [19, 97]. Therefore, various lines of evidence point to the cerebellum being involved in the modulation of central mechanisms linked to hedonic satiation as well as homeostatic regulation of feeding behaviour.

#### Cerebellum and Leptin-Mediated Appetite Regulation

Short-term feeding regulation limits food intake per meal, whilst long-term regulation dictates daily food intake [84, 86]. One circulating hormone that limits food intake in the short-term is cholecystokinin, which is secreted in the duodenum and induces satiety [83, 85, 110]. Circulating hormones regulating long-term appetite are insulin, which is secreted by the pancreas and governs glucose metabolism [111], and ghrelin, an appetitive stimulant secreted by the stomach [109, 112]. An additional hormone, leptin, is continuously secreted by adipose tissue [113, 114] and is an important regulator of appetite [113, 115, 116, 117], contributing to the process of meal termination [118, 119, 120].

Leptin is of particular interest to the role of the cerebellum in appetite regulation. High levels of leptin and leptin receptor expression have been reported in the rodent and human cerebellum [113], particularly in the cerebellar cortical granule cell layer but also in Purkinje cells and the lateral nucleus [121, 122]. High levels of cerebellar leptin have been associated with various physiological processes, for example during embryological development, the effect of leptin in the cerebellum is to promote survival, growth, and development of Purkinje cells [123]. Leptin has also been shown to facilitate NMDA receptor-mediated calcium influx in cultured cerebellar granule cells [124].

Low circulating levels of leptin are a hallmark of obesity [119, 125, 126] and deficiencies in leptin production, transport and signalling are known causes of increased weight [127, 128, 129, 130]. Leptin replacement therapies are therefore often used as a treatment for obesity [131]. Individuals with hereditary leptin deficiency have cerebellar activation within lobule VI and Crus I in response to food cues which decrease as patients undergo leptin-replacement therapy [132]. This suggests that leptin replacement counteracts abnormal levels of cerebellar activation in response to food cues associated with obesity [133]. Cerebellar activation patterns in relation to varying weights and volumes of adipose tissue might therefore contribute to changes in leptin signalling in overweight patients [87, 88].

It is well established that fluctuations in circulating leptin are detected by neurons in the hypothalamus which can increase or decrease appetitive behaviours accordingly [86, 134]. Given that the cerebellum is responsive to leptin replacement therapies used in obesity, this raises the interesting possibility that leptin also regulates appetite through its effects on the cerebellum. Further studies investigating the effects of leptin receptor activation on cerebellar neuronal activity would shed light on the mechanisms by which this may occur.

### Cerebellum in Mealtime Anticipation

The suprachiasmatic nucleus of the hypothalamus (SCN) is the circadian master clock [135] and has the primary responsibility for controlling and synchronising neural activity in relation to daylight cycles [136]. The basis of SCN function is differential expression of a number of genes that peak in expression at different times of the day, collectively termed Clock genes [137]. Clock gene expression can peak around anticipated mealtimes, thereby inducing hunger in a predictive, feed-forward manner [138, 139]. Secondary clocks also exist around the body; these are areas that express a number of Clock genes, and gene expression in these areas peaks with an approximate 6-h phase delay to the master SCN clock [138, 139].

Clock genes of particular interest for this review are period genes, *Per1* and *Per2* [137]. These are expressed in the granule cell layer and Purkinje cell layer of the cerebellar cortex and are involved in mediating food anticipatory activity around expected mealtimes [140, 141]. In food-restricted animals, expression of *Per 1* and *Per 2* in the cerebellum is phase-shifted to peak around expected mealtime, several hours earlier than the peak *Per 1* and *Per* 2 expression when animals have free access to food and therefore no set mealtime [142, 143]. Accordingly, depletion of Purkinje cells or disruption of the cerebellar circuitry can reduce food anticipatory activity [140, 141, 142]. Peaks in the cerebellar expression of Clock genes are delayed by several hours relative to the SCN, and it is likely this is controlled (perhaps indirectly) by the SCN [144].

In summary, the cerebellum has been shown to be involved in various mechanisms of appetite regulation, including key hormones and circadian rhythm genes, and cerebellar processing may differ between healthy and disordered eating. One outstanding question concerns how Clock genes, and their regulation, differ in those with disordered eating, as this could provide evidence for underlying genetic causes in such disorders. Similarly, there may also be genetic causes of differences in leptin receptor expression and responsivity in obesity.

## Cerebellar Reward Processing and Contributions to Over-Eating

Appetitive behaviours are governed by pleasurable sensations and desire of food, so it is important to consider the rewarding nature of eating [145]. The cerebellum is connected with reward centres in the brain including the VTA, striatum and neocortex [105, 146, 147, 148, 149] and outputs from the cerebellar lateral nucleus have been found to mediate hedonic aspects of satiation by increasing dopaminergic release in the VTA [97]. The cerebellum is likely therefore to be involved in assigning food with a reward value. This section will outline recent animal studies providing evidence that reward information is indeed processed by the cerebellum and the implications of abnormal cerebellar responses to food cues in over-eating disorders.

### Reward-Related Signalling in the Cerebellum

An increasing body of evidence suggests that different elements of reward-related information are encoded within the cerebellum. For example, in mice trained to perform a voluntary movement for reward, a population of cerebellar granule cells in lobules VIa, VIb and lobulus simplex were shown to respond to reward delivery or reward omission, whilst others encoded reward anticipation [150]. These response profiles developed over the learning period, suggesting that the cerebellum learns to predict reward delivery and adapt its responses based on experience. Granule cells receive synaptic input from mossy fibres (Fig. 1b), which are therefore likely to carry this information.

The other main input to the cerebellum is from climbing fibres (Fig. 1b), which also carry multiple types of information related to reward. Climbing fibre inputs to the cerebellar flocculus have been shown to encode reward size; in monkeys cued to the size of a reward in an eye movement task, climbing fibre activity increased in response to a large but not a small reward cue [151]. In the lateral cerebellum of mice, climbing fibres have also been shown to signal reward prediction (lobule simplex, Crus I and II) during learning, and can also signal reward delivery and omission [152].

In agreement with classical theories of cerebellar-dependent motor learning [153], reward omission information conveyed by climbing fibres may serve as an error signal which occurs when the outcome is unexpected. In support of this idea, climbing fibre responses to predictable rewards were suppressed during learning of a visuomotor integration task [154], and the phenomenon can also be generalised to the cerebellar mossy fibre-granule cell-parallel fibre system because reward-related error signals in PC simple spike responses diminish as monkeys learn a reward-association task [155]. Reward-based learning in the cerebellum would seem therefore to be driven by similar mechanisms as error-based motor learning, where the cerebellum learns to predict the expectation of a reward and an error signal occurs when an expected reward does not materialise.

### Cerebellum in Pathophysiological Cue Processing

In addition to evidence from animal models that the cerebellum processes rewarded-related cue information, imaging studies in humans suggest that this function is conserved across species. The cerebellum can learn to selectively respond to rewarding cues, including highly palatable food [156], and altered cue processing may underlie several pathophysiological states including over- and under-eating disorders (the latter are explored further in the section Cerebellar Contributions to Affective Aspects of Eating).

Activation of the anterior cerebellum (hemispheric lobule VI) and VTA is enhanced in overweight participants compared to healthy weight participants when exposed to cues for highly palatable foods [157]. Overweight individuals also find palatable foods more appealing when full compared to full healthy-weight individuals [133]. This is correlated with over-responsiveness in the cerebellum, as obese children show increased cerebellar activation in response to exposure to highly palatable foods once satiated, as compared to healthy weight children [158]. Participants with Prader-Willi syndrome have obesity characterised by dysfunctional reward processing circuits, including potential overactivation of subcortical reward circuitry and under activation of cortical inhibitory regions after eating [159]. Prader-Willi participants also show increased activation in regions of the cerebellum, likely corresponding to the cerebellar nuclei, in response to food cues post-meal compared to controls [97].

Changes in perception of hunger and satiety may be regulated by cerebellar connections with other reward centres in the brain. For example, neuromodulation of prefronto-cerebellar connections using transcranial direct current stimulation (tDCS) increased the desire to eat upon exposure to visual food cues [160], suggesting that disrupting this circuit could impair normal regulation of food intake. Decreased functional connectivity between the LH and the cerebellar cortex has also been reported in overweight compared to healthy-weight participants [161]. As the LH is involved in processing both physiological and pleasurable eating, this decreased connectivity could indicate that the cerebellar-LH connection contributes to food being perceived as less rewarding in obese participants, which may lead to overeating to obtain a similar reward value. Altered cerebellar-VTA connections are also associated with overeating in mice [97].

In summary, animal studies have demonstrated that reward information is processed in the cerebellum and that the cerebellum interacts with other reward centres [152, 154, 162]. Imaging studies have suggested that altered reward processing within the cerebellum could be a key contributor to the pathophysiological events of compulsive eating experienced by obese and overweight individuals. Understanding how the cerebellum learns pathological reward-related responses and how it could ‘re-learn’ healthier associations could provide insight to approaches aimed at preventing or treating overeating and obesity.

## Cerebellar Contributions to Affective Aspects of Eating

Appetite is influenced by affective state, and the cerebellum is a key node for affective processing through its connections with brain regions including the limbic system and prefrontal cortex [8, 9, 10, 12, 163, 164, 165, 166, 167, 168]. This section will discuss how cerebellar involvement in affective processing may be linked to appetite in both negative emotional states (stress and anxiety) and survival (fear and innate survival).

### The influence of Negative Emotions on Eating

Stress and anxiety strongly influence appetite; whilst anxiety is predominantly an appetite suppressant, stress can either suppress or stimulate appetite depending on the palatability of available food and whether the stressor is acute or chronic [23]. Stress has been linked with changes in weight and corresponding neural activity. Alterations in functional connectivity between the cerebellum and regions such as the LH and the hippocampus have been linked with a higher risk of weight gain and overeating in stressed participants [169, 170, 171, 172]. Stress can also interfere with dopaminergic signalling in the VTA [169, 172], which may change the way in which the cerebellum controls VTA dopamine release in relation to hedonic satiation [97].

The cerebellum has been linked to emotional processing disorders in which anxiety is commonly reported, including schizophrenia, autism and depression [173, 174, 175, 176, 177]. As well as affecting appetite in healthy weight individuals, anxiety is also strongly related to eating disorders including anorexia nervosa. Imaging studies have shown an altered cerebellar network connectivity in anorexia nervosa patients compared to healthy controls, in particular increased activity within the vermis [178, 179, 180]. The disorder is associated with altered food-cue processing and decreased appetite upon presentation of food cues [172, 181, 182]. This contrasts with the cerebellar over-responsiveness to food cues described in obese participants [97, 157].

The cerebellum is therefore involved in processing negative emotions as well as the regulation of appetite (see Cerebellar Contributions to Appetite Control) which has been shown to vary in several affective disorders [23, 172, 183]. The cerebellum may have an impaired predictive ability in affective conditions which could alter the perceived reward value of foods and impact the anticipation of appropriate times to eat. This could lead to fluctuations in the volume of food consumed at individual meals and increase or decrease number of meals over a longer term, leading to changes in weight.

### Cerebellum and Survival

Cerebellar activation has been associated with survival functions [171, 184, 185], for example cerebellar activation, particularly within the vermis and anterior paravermis, has been shown in response to hypercapnia (hunger for air) and thirst [186, 187, 188, 189, 190, 191]. It may therefore be hypothesised that the cerebellum drives appetitive functions in response to an innate need to replenish energy stores. The cerebellar vermis is a common link between affective disorders, survival networks, homeostatic functions, and hunger [81, 192, 193, 194], indicating that it could be a key node which regulates affective influences on appetite. It remains to be determined if separate vermal regions are related to each of these functions, or whether there are overlapping roles of individual regions.

Associative fear learning, like reward-based learning and classical cerebellar motor learning (e.g., eyeblink conditioning), can be driven by prediction errors, and negative prediction errors drive the extinction of conditioned fear responses [40, 195, 196, 197]. A universal mechanism of cerebellar learning, based on prediction, may therefore apply across multiple types of behaviour, including eating. In this case, the cerebellum may regulate appetite by integrating metabolic (leptin and other circulating factors), sensory (food cues), and proprioceptive (hedonic satiation) factors in a predictive error-correction manner.

## Cerebellum and Prediction

The traditional error-based model of cerebellar learning uses feed forward predictions (internal models) about the sensory outcomes of movement and compares these against movement-related sensory feedback to supervise error correction, thereby improving performance accuracy (for more details see [198]. An extension of this theory is to consider the cerebellum as a ‘prediction machine’ [1, 2, 40, 41]. This extends cerebellar involvement in specific forms of motor learning, for example eyeblink conditioning, to more complex neocortical prediction paradigms involving interactions between multiple brain regions [199, 200, 201, 202]. For example, preparatory changes in firing rate have been reported in both the medial [203] and lateral cerebellar nuclei in mice [204], and inhibiting these regions has been shown to disrupt preparatory activity in the anterolateral motor cortex thereby impairing motor planning [203]. This suggests that the predictive capabilities of the cerebellum influence neocortical processing and could underpin its involvement in cognitive processing.

Prediction and feedback underlie many elements of eating described in this review, as food consumption is influenced by expectation. As well as short- and long-term regulation of appetite which works in a feedback fashion, appetite can also be regulated in a feed forward manner by circadian clocks, for example becoming hungry in anticipation of a meal [15]. The sight and smell of food will generate an internal expectation of what that food will taste like, and sensory information upon eating that food will provide feedback to match or contradict that expectation. As outlined in the section Cerebellar Reward Processing and Contributions to Over-Eating, evidence is accumulating that the cerebellum encodes information about reward prediction and is likely to do so for other properties of reward, such as preferred taste. This internal prediction may be relayed to the hypothalamus to influence satiety-inducing neural circuits depending upon expectation and outcome.

This predictive model may explain disordered eating (both under- and over-eating), where often the feedback signals are maladaptive. For example, overweight individuals are more responsive to food cues and have altered satiation signalling. This lack of accurate feedback could prevent the learning of healthy eating behaviours, and affective or rewarding elements may override basic homeostatic regulation, in line with evidence that prediction errors are also present in the emotional domain [195]. We therefore propose that the overarching role of the cerebellum in eating is to generate a behaviourally relevant response to prediction errors which arise from a variety of domains including homeostatic, motor, reward, and affective signals.

The ability of the cerebellum to contribute to such a variety of functions related to eating stems from its extensive interconnectivity with other brain regions, as explored in this review. Altered connectivity within this brain-wide network may contribute to disordered eating. It follows that manipulating this network to restore altered connectivity may provide a therapeutic approach for disorders associated with eating. The feasibility of such an approach is now possible with the development of non-invasive neuromodulatory techniques including tDCS, transcranial alternating current stimulation and transcranial magnetic stimulation [205, 206]. Applying these techniques to the cerebellum has been shown to alter cerebellar connectivity with cognitive regions of the cerebral cortex [207], improve performance in motor tasks likely via altering excitability in motor cortical regions [208], and improve cognitive performance in patients with bipolar disorder [209]. As outlined in the section Cerebellum in Pathophysiological Cue Processing, increasing prefrontal cortical activity and decreasing cerebellar activity using tDCS can increase the propensity to eat [160], showing that such approaches can directly influence eating behaviour. Therefore, non-invasive stimulation of the cerebellum would seem to be a promising approach to explore in future efforts to treat pathological eating processes.

## Summary

**Fig. 3:**
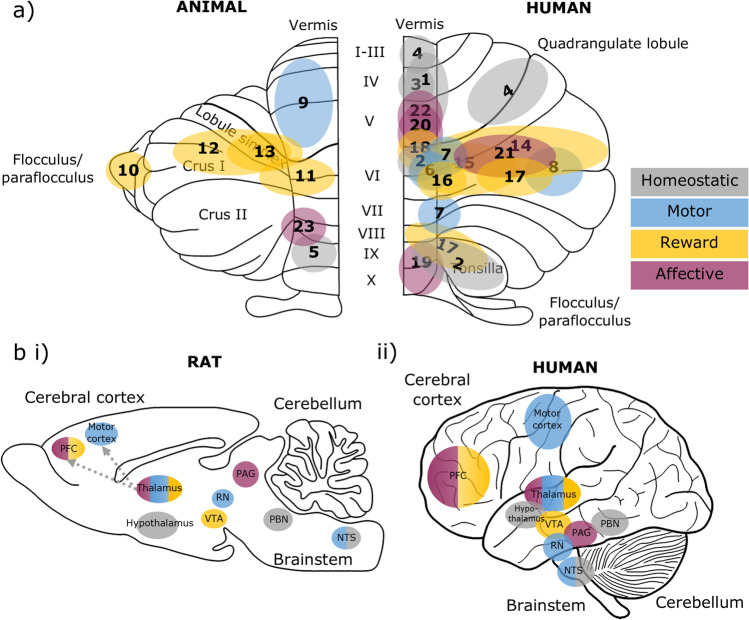
Cerebellar and brain-wide networks involved in eating behaviours. **a** Cerebellar regions shown to be involved in homeostatic (grey), motor (blue), reward (yellow) and affective (purple) aspects of eating. Animal studies are depicted on an outline of the rat cerebellum on the left, and human studies are shown on the right. The numbers correspond to the studies detailed in Table 1. **b** The cerebellum has connections with brain regions contributing to homeostatic (grey), motor (blue), reward (yellow) and affective (purple) domains of eating behaviours, depicted in a (i) rat and (ii) human brain outline. Connections may be direct or indirect; the latter is the case for cerebello-thalamo-cortical pathways. We propose that the cerebellum has a unifying role via prediction signals which contributes to each of these components. Note that this diagram is not comprehensive, but represents key structures discussed in this review. PFC, prefrontal cortex; VTA, ventral tegmental area; RN, red nucleus; PAG, periaqueductal grey; PBN, parabrachial nucleus; NTS. nucleus tractus solitaries

This review has outlined cerebellar contributions to the motor, homeostatic, rewarding, and affective aspects of eating. The regions of the cerebellar cortex involved in each of these domains are summarised in Fig. 3a (and corresponding Table 1). The distribution of cerebellar contributions suggests that the medial parts of the cerebellum, which are phylogenetically the oldest, largely contribute to the homeostatic, motor and survival aspects of eating. In contrast, the phylogenetically newer regions of the cerebellum, the hemispheres, are related to higher-order aspects of eating, including reward processing and associative learning (both appetitive and affective). Cerebellar contributions to such a wide range of functions can be achieved through its widespread connections with other brain regions involved in each function, as summarised in Fig. 3b.

**Table 1:**
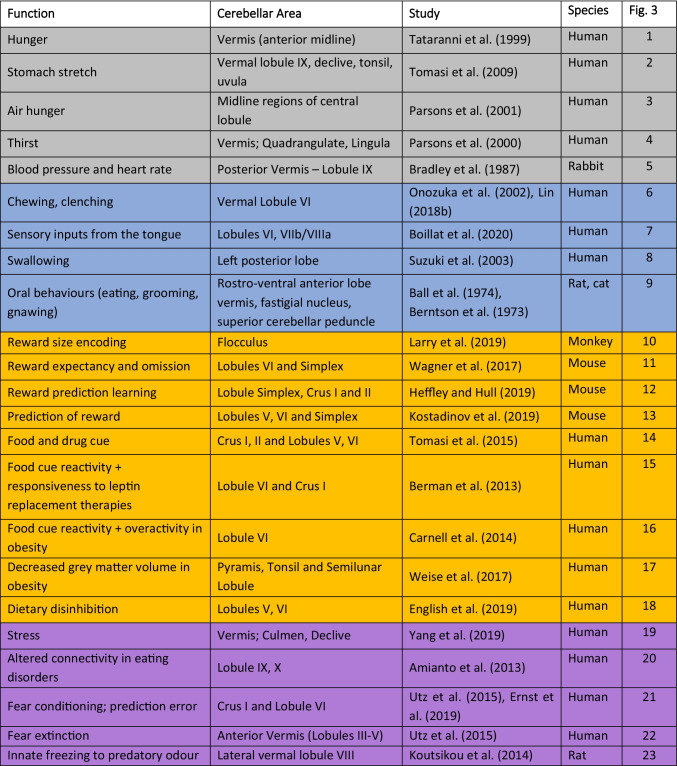
Summary of cerebellar regions involved in different aspects of eating behaviours. Colours correspond to homeostatic (grey), motor (blue), reward (yellow) and affective (purple) functions. These areas are depicted on a cerebellar outline in Fig. 3a

Evidence for a role of the cerebellum in feeding behaviour exists at a molecular (e.g., via leptin signalling), anatomical (neuronal connections with regions including the hypothalamus, PBN and NTS), and functional level (imaging studies showing activation during hunger and in the presence of pleasant taste sensations). The cerebellum is involved in processing reward and associated cues which may induce cravings and the false sensations of hunger. In obesity, this could contribute to overeating by both inhibiting the sensation of satiety and being hyperreactive to food cue processing. On the other hand, underactive food cue processing impacts appetite and, together with the involvement of the cerebellum in affective processing, could contribute to the symptomatology of eating disorders. We propose that the cerebellum may control each of these elements in its role as a prediction machine. Future experiments could investigate this by examining predictive behaviours, such as mealtime or reward anticipation, and abnormal eating patterns (under- or over-eating) following targeted cerebellar inactivation in animal models and observing changes in eating habits of cerebellar patients.

A number of outstanding questions remain, including:

- Is the same cerebellar computation carried out when predicting the different types of behaviour involved in eating (motor, homeostatic, reward, affective), or is it region-specific? Could it depend on the time course of individual predictions, given that these behaviours happen over different time scales? For example, motor control of swallowing versus circadian control of appetite.
- Could genetic predispositions to eating-related disorders directly affect gene expression patterns in the cerebellum?
- Can we truly separate the motor, homeostatic, cognitive, and affective aspects of feeding behaviours? Are all feeding networks likely to be involved in multiple domains to some extent?
- Could non-invasive stimulation techniques be useful therapeutic approaches for treatment of disordered eating?
